# IFN-λ Inhibits MiR-122 Transcription through a Stat3-HNF4α Inflammatory Feedback Loop in an IFN-α Resistant HCV Cell Culture System

**DOI:** 10.1371/journal.pone.0141655

**Published:** 2015-12-11

**Authors:** Fatma Aboulnasr, Sidhartha Hazari, Satyam Nayak, Partha K. Chandra, Rajesh Panigrahi, Pauline Ferraris, Srinivas Chava, Ramazan Kurt, Kyongsub Song, Asha Dash, Luis A. Balart, Robert F. Garry, Tong Wu, Srikanta Dash

**Affiliations:** 1 Pathology and Laboratory Medicine, Tulane University School of Medicine, 1430 Tulane Avenue, New Orleans, LA-70112, United States of America; 2 Department of Medicine, Division of Gastroenterology and Hepatology; 3 Microbiology and Immunology Tulane University School of Medicine, 1430 Tulane Avenue, New Orleans, LA-70112, United States of America; University of Sydney, AUSTRALIA

## Abstract

**Background:**

HCV replication in persistently infected cell culture remains resistant to IFN-α/RBV combination treatment, whereas IFN-λ1 induces viral clearance. The antiviral mechanisms by which IFN-λ1 induces sustained HCV clearance have not been determined.

**Aim:**

To investigate the mechanisms by which IFN-λ clears HCV replication in an HCV cell culture model.

**Methods:**

IFN-α sensitive (S3-GFP) and resistant (R4-GFP) cells were treated with equivalent concentrations of either IFN-α or IFN-λ. The relative antiviral effects of IFN-α and IFN-λ1 were compared by measuring the HCV replication, quantification of HCV-GFP expression by flow cytometry, and viral RNA levels by real time RT-PCR. Activation of Jak-Stat signaling, interferon stimulated gene (ISG) expression, and miRNA-122 transcription in S3-GFP and R4-GFP cells were examined.

**Results:**

We have shown that IFN-λ1 induces HCV clearance in IFN-α resistant and sensitive replicon cell lines in a dose dependent manner through Jak-Stat signaling, and induces STAT 1 and STAT 2 activation, ISRE-luciferase promoter activation and ISG expression. Stat 3 activation is also involved in IFN-λ1 induced antiviral activity in HCV cell culture. IFN-λ1 induced Stat 3 phosphorylation reduces the expression of hepatocyte nuclear factor 4 alpha (HNF4α) through miR-24 in R4-GFP cells. Reduced expression of HNF4α is associated with decreased expression of miR-122 resulting in an anti-HCV effect. Northern blot analysis confirms that IFN-λ1 reduces miR-122 levels in R4-GFP cells. Our results indicate that IFN-λ1 activates the Stat 3-HNF4α feedback inflammatory loop to inhibit miR-122 transcription in HCV cell culture.

**Conclusions:**

In addition to the classical Jak–Stat antiviral signaling pathway, IFN-λ1 inhibits HCV replication through the suppression of miRNA-122 transcription via an inflammatory Stat 3–HNF4α feedback loop. Inflammatory feedback circuits activated by IFNs during chronic inflammation expose non-responders to the risk of hepatocellular carcinoma.

## Introduction

Hepatitis C virus (HCV) infection is a major public health concern, affecting an estimated 170 million people worldwide [1]. The majority of individuals infected with HCV cannot clear the virus naturally, and progress to chronic infection [2]. Chronic HCV infection is the major cause of liver cirrhosis, end-stage liver disease, and hepatocellular carcinoma [3]. Moreover, treatment of chronic infection with interferon (IFN-α) plus ribavirin (RBV) combination antiviral therapy has been unsatisfactory, showing a success rate of ~50% [4]. Very recently, the cure rate of HCV has improved significantly due to the development of novel direct-acting antiviral agents (DAAs) [5, 6].

It has been shown that genetic polymorphism of the IFN-λ gene is strongly associated with success of HCV antiviral treatment, and is a strong predictor of hepatic inflammation and liver disease progression [7–11]. Genetic variations within the interleukin (IL)-28B promoter are strongly associated with the outcome of HCV treatment using a combination of IFN-α plus RBV [12–14, 15, 16, 17]. Patients with the IL-28B C/C genotype rs12979860 show 2–5 times better HCV clearance by IFN-α plus RBV treatment than do patients subject to the same treatment but with the T/T genotype. Chronic HCV patients with activated expression of IFN-stimulated genes (ISGs) in the liver have also shown poor response to IFN-α plus RBV treatment. An important recent discovery indicates that patients who express functional IFNλ4 in the liver show impaired clearance by IFN-α plus RBV treatment, as compared to individuals who express a non-functional frame-shift variant of the IFNλ4 gene [18, 19]. Intrahepatic production of IFNλ4 is responsible for transcriptional activation of ISGs and HCV clearance [18], which strongly supports the importance of the IFN-λ axis for driving antiviral defense mechanisms in cases of chronic HCV infection.

Genetic polymorphism in IFN-λ is also a strong predictor of hepatic inflammation and fibrosis in patients with viral and non-viral liver disease [7]. Type III IFN levels are elevated in patients with chronic liver disease on account of host defense mechanisms [20]. However, the role of the IFN-λ axis in modulating the host inflammatory response in chronic HCV infection is not well understood. In the liver, microRNA-122 (miR-122) regulates hepatocyte growth, lipid metabolism, and neoplastic transformation; miR-122 also binds to HCV internal ribosome entry sites (IRESs) in infected hepatocytes, and a miR-122 inhibitor has been shown to induce HCV clearance in chimpanzees [21]. A recent report confirms that IFN-λ antiviral mechanisms involve inhibition of miR-122 expression in hepatocytes [22]. Serum miR-122 levels have been shown to positively correlate with positive outcomes of IFN-α plus RBV treatment of individuals with the IL-28B genotype, indicating a possible causal connection between IFN-λ and miR-122 expression [23]. The transcription of miR-122 in the liver is regulated by hepatic nuclear factor 4 alpha (HNF4α) [24], which supports the importance of type III IFN in the pathogenesis of chronic HCV infection.

Interferons play an important role in the defense against a wide variety of viral infections, inflammation, and cancers. They are classified into three distinct types based on amino acid sequence homologies and interactions with cell surface receptors: type I IFNs (IFN-α and IFN-β) bind to IFN-α receptors, type II IFNs (IFN-γ) bind to IFN-γ receptors, and type III IFNs, which include IFN-λ1 (IL-29), IFN-λ2 (IL-28A), and IFN-λ3 (IL-28B), bind to IFN-λ receptors. Although type I and type III IFNs bind to two separate receptors, they both mediate the same antiviral signaling, primarily through activation of the Janus kinase–signal transducer and activator of transcription (Jak–Stat) pathway [25]; IFN-α binds to the IFN-α/β receptor complex (IFNAR1/2), leading to phosphorylation of Stat 1 and Stat 2 by the tyrosine kinases Jak 1 and Tyk 2. The phosphorylated Stats associate with interferon regulatory factor (IRF) 9 to form the ISG factor 3 (ISGF 3) transcriptional complex, which translocates to the nucleus where it binds to the promoter region of all ISGs and then initiates antiviral gene transcription. Type III IFNs are able to inhibit HCV virus replication in a manner similar to IFN-α through activation of the Jak–Stat signaling pathway and induction of ISGs [25]. The biological activities of these two IFNs appear to be different at the level of antiviral activity for the duration of Jak–Stat activation [26].

The IFN-λ molecule is a recently discovered member of the IFN-family. The role of this cytokine in antiviral mechanisms is not yet well understood. A series of studies conducted in our laboratory have shown that HCV-induced endoplasmic reticulum (ER) stress and the autophagy response inhibit the expression of IFN-α receptor 1 (IFNAR 1), which explains the prevention of IFN-α antiviral activity [27–29]. The expression of IFN-λ receptors is not altered in HCV-infected cell cultures, which is why IFN-λ efficiently inhibits HCV replication. We observed robust antiviral effects of IFN-λ in IFN-α-resistant cells. Thus, the present study was conducted to understand the antiviral mechanism by which IFN-λ inhibits HCV replication in IFN-α-resistant HCV cell cultures. We demonstrate in this study that IFN-λ is capable of effectively inhibiting HCV replication in a cell culture model that is resistant to the action of IFN-α through activation of the Jak–Stat signaling pathway and induction of ISGs. We also found that IFN-λ activates Stat 3 phosphorylation in a concentration-dependent manner, and decreases miR-122 transcription through the Stat 3–HNF4α inflammatory feedback loop. This study provides a novel antiviral mechanism by which IFN-λ clears HCV replication, dominantly when IFN-α signaling is impaired.

## Materials and Methods

### Cell culture and chemicals

We used IFN-α sensitive (S3-GFP) and IFN-α resistant cell lines (R4-GFP) previously developed in our laboratory [30]. These cells were maintained in Dulbecco’s Modified Eagle’s Medium (DMEM) supplemented with 2mM L-glutamine, sodium pyruvate, nonessential amino acids, 100U/mL penicillin, 100mg/mL streptomycin, and 10% fetal bovine serum supplemented with G-418 (1mg/mL). These cells were maintained long-term in growth medium containing G-418 (1mg/ml) by splitting at a ratio of 1:5 at three-day intervals. Huh-7.5 cells were obtained from Charles M. Rice (Rockefeller University, New York). The Huh 7.5 cell line was maintained in Dulbecco’s Modified Eagle’s Medium (DMEM) supplemented with 2 mM L-glutamine, sodium pyruvate, nonessential amino acids, 100U/mL penicillin, 100mg/mL streptomycin, and 10% fetal bovine serum. The persistently infected Huh 7.5 cell culture system was developed in our laboratory [27]. Recombinant human IFN-α2b (Intron A) was purchased from Schering Plough, Kenilworth, NJ. Recombinant human IFN-λ1 was obtained from Peprotech Rocky Hill, NJ. pISRE-luciferase was provided by Stephen Goodbourn, St. George's Hospital and Medical School, University of London, London, UK. Antibodies against Stat 1, pStat 1, Stat 2, pStat 2, Stat 3, pStat 3, Jak 1, p 38, pJNK1/2,pERK1/2, pAKT, pPKC and β-actin were obtained from Cell Signaling (Beverly, MA). Antibodies to GAPDH, MX 1, OAS 1 and HNF4α were purchased from Santa Cruz Biotechnologies. Antibody for PKR was purchased from Abcam. Stattic a STAT 3 inhibitor was obtained from Santa Cruz laboratory. Jak inhibitor (Pyridone-6) was obtained from Calbiochem, San Diego, CA. MiR-122 mimic was purchased from Qiagen, Valencia, CA. The Renilla luciferase reporter based pJFH1-ΔV3-Rluc clone has been described previously [27].

### Western blotting

To measure phosphorylated proteins, S3-GFP and R4-GFP cells were treated with IFN-α or IFN-λ for 30 minutes before harvesting. Cells were harvested by the treatment of trypsin-EDTA. Cells were lysed in ice-cold lysis buffer (50mM Tris HCl pH 8.0, 140 mM NaCl, 1.5 mM MgCl2, 0.5% NP-40 with complete protease inhibitor from Invitrogen) for 10 minutes in ice (about 1X10^6^ cells/200 μL). Whole cells and cell debris were pelleted by low speed centrifugation and cleared supernatants were transferred to a new tube. Protein concentration was determined by Nanodrop (Thermo Scientific). Samples were boiled for 10 minutes at 80°C in the presence of 1X SDS-PAGE-loading buffer (250mM Tris-HCL pH 6.8, 40% glycerol, 8% SDS, 0.57M β-mercaptoethanol, 0.12% bromophenol blue). 20μg of protein was loaded onto 12% SDS-PAGE and transferred into a nitrocellulose membrane (Hybond, Amersham Biosciences). The membrane was blocked using 5% blotting-grade milk powder (Biorad, Hercules, CA), for one hour then incubated with primary antibody. After overnight incubation, the antigen-antibody complex was visualized with HRP-conjugated goat anti-rabbit or anti-mouse IgG and the ECL detection system (Invitrogen, Pierce, Amersham).

### RNA extraction and ISG quantitation by real-time RT-PCR

S3-GFP and R4-GFP or persistently HCV infected Huh-7.5 were seeded at a density of 1x10^6^ cells in 10-cm plate and incubated for 24 hours. The next day, cells were treated with IFN-α (10–1000 UI/mL) or IFN-λ1 (1-100ng/mL) for 24hours. Cells were harvested and lysed and RNA isolation was performed using the Pure Link ^TM^ RNA mini kit (Ambion). One microgram of total RNA was mixed with 5μM of antisense primer and cDNA was synthesized for one hour at 42°C. The reverse transcription for each mRNA was carried out using a standard method established in our laboratory, except for the gene specific antisense primer. The qPCR assay was carried out in 20μL containing 10μL of iQ supermix (Bio-Rad laboratories inc.), 5 μM of each primer, and 4μl of cDNA product obtained from the RT reaction. Each reaction was run in triplicate. The oligonucleotide sequences for sense and antisense primer used for quantification of PKR, MxA, and OAS1 are listed in **Table 1**. The qPCR was performed using the CFX96 C1000 Real-Time Instrument Thermal Cycler (BioRad, Hercules, CA). The amplification was carried out using the following PCR program: First cycle at 50°C for 2 min, then 95°C for 8 min, followed by an additional 40 cycles. Each PCR cycle included a denaturation step at 95°C for 30 seconds, then an annealing and extension step at 60°C for one minute. Data acquisition and analysis were performed using CFX manager software (Bio-Rad, Hercules, CA). For quantification of HCV RNA, RNA was isolated using the GITC method, then 2 μg of cellular RNA were reverse transcribed to cDNA using the antisense primer to the HCV 5’ UTR listed in the table below. 4μL of the cDNAs were added to a 20-μL reaction containing the iQ supermix (Bio-Rad Laboratories, Hercules, CA) and 5 μM/L of each primer and the probe (**Table 1**). The amplification and data analysis were performed using the CFX96 real-time PCR system with CFX Manager Software version 1.0 (Bio-Rad Laboratories).

**Table 1:** 

Primer	Sequence
HCV 5’UTR	F: TCTTCACGCAGAAAGCGTCTA
	R: CGGTTCCGCAGACCACTATG
MXA	F:GCCGGCTGTGGATATGCTA
	R:TTTATCGAAACATCTGTGAAAGCAA
OAS1	F:AGAAGGCAGCTCACGAAACC
	R: CCACCACCCAAGTTTCCTGTA
PKR	F:TGGAAAGCGAACAAGGAGTAAG
	R: CCATCCCGTAGGTCTGTGAA
HNF4-α	F: TGTCCCGACAGATCACCTC
	R: CACTCAACGAGAACCAGCAG
GAPDH	F: AGA ACA TCA TCC CTG CAT CC
	R: AGT TGC TGT TGA AGT CGC
hsa-miR-122-5p	5’UGGAGUGUGACAAUGGUGUUUG
hsa-miR-24-3p	5'UGGCUCAGUUCAGCAGGAACAG

### MicroRNA Array

R4-GFP cells were seeded at 1 X 10^6^ cells/100 mm dish and treated with IFN-λ for 6 hours. MicroRNA was extracted from IFN-λ treated and untreated cultures using PurelinkTM miRNA Isolation Kit (Cat# K1570-01, Invitrogen) according to the manufacturer’s instructions. In order to compare the profiles of miRNA expression in these samples, we employed the miRCURY LNA microRNA Array, 6th gen-hsa, mmu & rno (Exiqon, Cat# 208400) (Tulane Primate Center, Covington). This array platform allows a simultaneous screening of the expression of all known human miRNA. Samples extracted from the control (S) cells were labeled with Cy3, while those from the experimental (R) cells were labeled with Cy5, using the mercury LNA miRNA labeling kit (Exiqon). Cyanine-labeled samples were then mixed together in equivalent concentrations and hybridized to the array overnight at 55°C in a rotary chamber. Slides were scanned on a dual confocal Axon GenePix 4000B scanner (Molecular Devices) using GenePix version 6.2 software and the raw data was extracted. A stringent set of criteria was applied to remove background and highly variable data from consideration. The remaining data was log2 transformed and normalized using Locally Weighted Scatter-plot Smoothing in Spotfire S+, to remove intensity-specific bias. MicroRNAs were considered to be differentially expressed if they exhibited a 2-fold perturbation in expression magnitude.

### Quantification of miRNA

For quantification of miR-122, 250ng of total cellular RNA including miRNA was isolated using the miRNAeasy kit from Qiagen. The reverse transcription reaction was performed using the miScript II RT Kit also from Qiagen. The cDNA was diluted by adding 200μL of RNAse free water, and 2.5μL were added to a 25μL reaction containing 12.5μL of the QuantiTect SYBR Green PCR Master Mix from Qiagen, in addition to 1μL of the miScript Universal Primer and 1 μL of the miscript primer for miR-122. The miRNA RNU6-2_11 was used as a control. Sequences of the primers are listed below in **Table 1**.

### Northern Blotting

MicroRNA was isolated from IFN-λ treated and untreated R4-GFP cells using the miRNAeasy kit (Qiagen). Expression microRNA in the R4-GFP cell line was examined by Northern blot analysis. A total of 15μg of the purified RNA along with 10μL formamide, 3.5μL formaldehyde, 2μL 10X MOPS, 2.5μL 10X loading dye and 1μL ethidium bromide in a final volume of 20μL. Prior to the loading, samples were heated to 95°C for 5 min and kept on ice. The samples were loaded on a 1% agarose formaldehyde gel and run for 1.5 hours at 60V. Wet transfer of microRNA onto a nylon membrane (BIORAD) was accomplised overnight at room temperature using 20X SSC. The next day, the membrane was washed in 1XSSC solution, UV cross-linked and pre-hybridized for 3 hours at 50°C. The membranes were hybridized using g-32P labeled oligonucleotide probes. The probes were radiolabeled using T4 kinase with γ-32P-αTP (Invitrogen). The sequence for the miR-122 oligonucleotide probe used was 5-αCAAACACCATTGTCACACTCCA-3 and the sequence for miRNA U6 probes used was 5-TATGGAACGCTTCACGAATTTGC-3. The membrane was hybridized with the probe overnight at 50°C. The next day the membrane was washed, exposed to Kodak X-ray film overnight and developed.

### DNA pull down assay

R4-GFP cells were treated with IFN-α or IFN-λ for 5 hours. The cell pellets were lysed by the addition of 200μL of HKMG buffer (10 mM HEPES, pH 7.9, 100 mM KCl, 5 mM MgCl2, 10% glycerol, and 0.5% NP-40 with protease inhibitors) followed by sonication for 3 cycles of 20 seconds each. Cell lysates were then centrifuged for 15 minutes at 12,000rpm. The clear supernatant was transferred to a clean tube and precleared by incubation with streptavidin-agarose beads (Thermo Fisher Scientific) for 1 hour at 4°C. The supernatant was then incubated overnight with 1 μg of biotinylated double-stranded oligonucleotide and 10μg poly (dI-dC) (Thermo Fisher Scientific) at 4°C. The oligonucleotide sequences are: 5’-Biotin-TGACCGGTGACTC-3’ (first DR1); and 5’-Biotin-TGGCCTAAGGTCG-3’ (second DR1). TEN buffers were used to anneal the biotinylated oligonucleotides and their complementary strands. DNA-protein combination was collected by incubation with streptavidin-agarose beads at 4°C for 1 hour. 100 μL of HKMG buffer was used to wash the beads four times and the samples were boiled at 95°C for 5 minutes in SDS-sample buffer prior to SDS-PAGE and Western blotting with specific antibodies.

### Nuclear Translocation Assay

Huh-7.5 cells were split into a 12-well cell culture plate (Thermo Fisher Scientific) at a density of 2 × 10^5^ cells per well. The next day, cells were transfected with 0.5μg of STAT3-GFP plasmid DNA using Turbofect (Thermo Fisher Scientific) transfection reagent. After 48 hours, STAT3-GFP transfected cells were treated with 1000 IU/mL IFN-α or 100ng/mL IFN-λ. 1 hour after treatment the nuclei were stained using DAPI (Southern Biotech, Bermingham,AL), and the translocation of GFP was monitored using fluorescence microscopy (Olympus TH4-100, Tokyo, Japan).

### HNF4α 3’UTR-Luciferase assay

S3-GFP and R4-GFP cells were plated in 6 well plates. Next day they were transfected with of miR-24-3P mimic (30pmole) (Qiagen), or a control oligonucleotide (Qiagen) using Lipofectamine RNAmaxi (Life technologies). After 48 hours, cells were transfected with 10ng of a luciferase reporter plasmid harboring the 3’ UTR of HNF4α (pRL-CON1-3180, Addgene #31445) or a control plasmid EGFP-Luc. Cells were harvested after 24 hours, lysed and the luciferase activity was measured.

### Promoter based luciferase reporter assays

S3-GFP and R4-GFP were seeded in a 24 well plate at a density of 1X10^4^ cells per well. After 20 hours 500ng of pRL-TK and miR-122 promoter plasmid p-(-5.7/-3.8 k) luciferase were co- transfected into each well using Turbofect. 24 hours after transfection cells were treated with 1000 IU/mL IFN-α or 100ng/mL IFN-λ or PBS for 6 hours. For the IFN-β promoter 500 ng of plasmid ISRE-Luc or 500 ng of EGFP-Luc were transfected. The cells were then treated with 1000 IU/mL IFN-α or 100ng/mL IFN-λ or PBS for 24 hours. The cells were collected and lysed using 100μL of passive lysis buffer. Luciferase activity of cell lysates was measured using a luciferase assay system kit (Promega, Madison, WI). Protein concentration was measured using a NanoDrop spectrophotometer (Thermo Fisher Scientific) and the luciferase activity was normalized per microgram of protein.

### Statistical Analysis

All measurements were made at least in triplicate. All results were expressed as mean ± SE (standard error). Comparison between two groups was performed with a Student’s t-test. To compare means within groups we performed one factor analysis of variance (ANOVA) using the GraphPad Prism software. We assumed that all measurements have normal probability distributions, which is expected for these types of data. The ANOVA analysis was significant when p≤0.05.

## Results

### IFN-λ inhibits HCV replication in persistently infected cells and IFN-α-resistant sub-genomic replicon cell culture models

**Fig 1:**
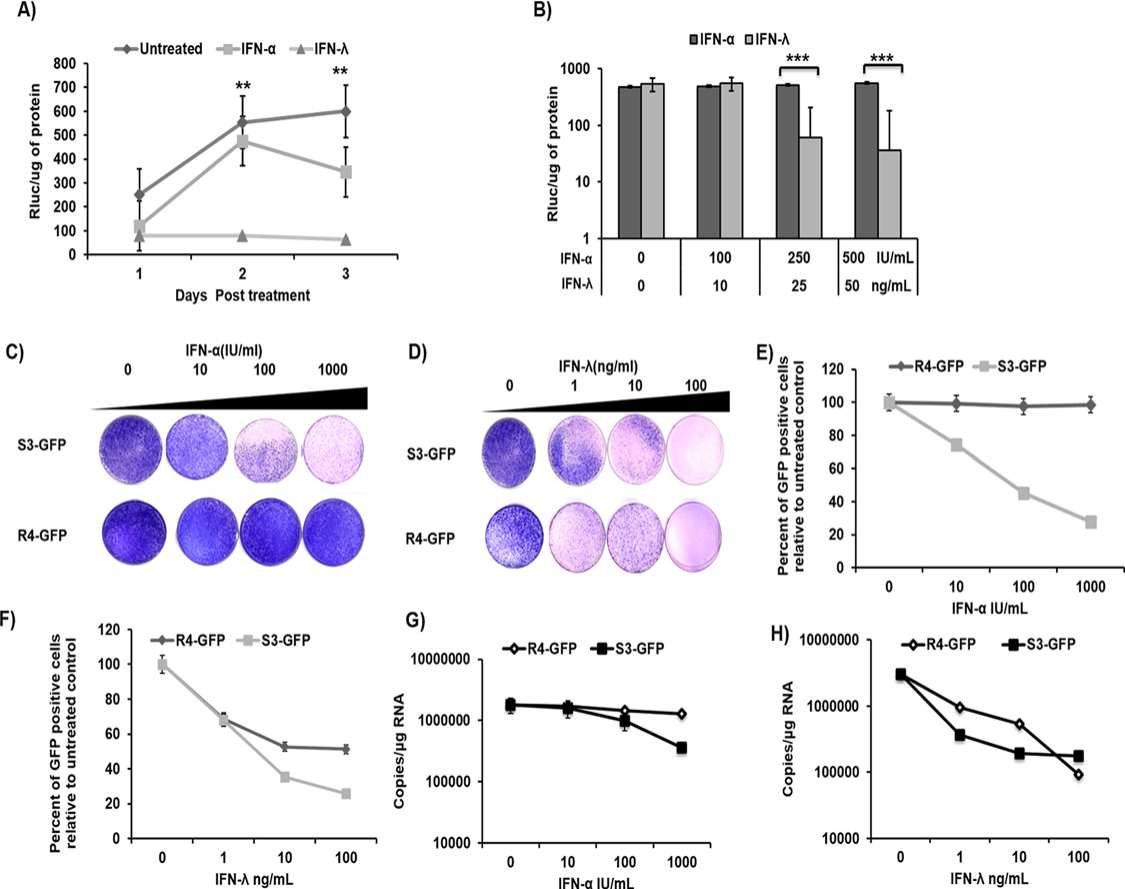
Sustained antiviral activity of IFN-λ HCV cell culture. **(A)** Persistently infected HCV (JFH1ΔV3-Rluc) cultures were treated with equivalent concentrations of IFN-λ1 (25 ng/ml) or IFN-α (250 IU/mL). Aliquot of infected cells were collected at every 24 hours and HCV replication was measured by *Renilla* luciferase activity. **(B)** Persistently infected HCV cultures were treated with increasing concentrations of IFN-α (100–500 I.U/mL) or IFN-λ (10-50ng/mL), HCV replication was measured at 24 hours. **(C)** R4-GFP and S3-GFP cells (2X10^5^/ 10-cm plate) were treated with IFN-α (10,100 or 1000 I.U/ml) in growth media supplemented with G-418 (1000ng/mL) for 6 weeks. The success of antiviral treatment was determined by the decrease in the number of the resistant colony. **(D)** The colony assay in the presence of IFN-λ (1, 10, or 100 ng/ml) treatment of S3-GFP and R4-GFP cells. (**E)** Quantification of HCV-GFP fusion protein by flow cytometry of S3-GFP and R4-GFP cells after IFN-α treatment. **(F)** Quantification of HCV-GFP fusion protein by flow cytometry of S3-GFP and R4-GFP cells after IFN-λ treatment. **(G)** HCV positive strand RNA levels in S3-GFP and R4-GFP cells by real-time RT-PCR after IFN-α treatment. **(H)** HCV positive strand RNA levels in S3-GFP and R4-GFP cells by real-time RT-PCR after IFN-λ treatment, **p≤0.01, ***p≤0.001.

This study investigated the mechanism of sustained inhibition of HCV replication in cell cultures by IFN-λ1 as compared to IFN-α. Persistently infected HCV cultures were treated with equivalent concentrations of IFN-α or IFN-λ1, and antiviral activities were compared by measurements of HCV replication via levels of Renilla luciferase (RLuc). We found that inhibition of HCV replication by IFN-λ1 was more significant in persistently infected cultures as compared to inhibition by IFN-α. The results are consistent with our previous findings that indicate that IFN-λ1 inhibits HCV replication in persistently infected cultures in a time- and dose-dependent manner **(Fig 1A and 1B)**. Concentrations of IFN-α of 100, 250, and 500 IU/ml were equivalent to concentrations of IFN-λ1 of 10, 25, and 50 ng/mL, respectively [27]. Only 70% of the cells expressed the HCV core protein, indicating that down-regulation of IFNAR1 may not be uniform in HCV-infected hepatic Huh-7.5 cell culture [27]. In this study, we used an IFN-α resistant sub-genomic replicon cell line previously described in [30]. This model was used to avoid the possibility of uneven down-regulation of IFNAR 1 in the infected culture (which could affect our data analysis) and thus to investigate the IFN-λ-specific antiviral mechanism. To verify that the IFN-response was different in the two cell lines, S3-GFP and R4-GFP cells in the culture were treated with increasing doses of IFN-λ1 (1–100 ng/mL) or IFN-α (10–1000 IU/mL) for 72 h. The antiviral effects were compared using assays that measured sub-genomic RNA replication (G-418 resistant colony assay) (**Fig 1C and 1D**), quantification of viral proteins (by flow analysis of green fluorescent protein; GFP) (**Fig 1E and 1F**), and viral RNA levels by real-time reverse transcription-polymerase chain reaction (RT-PCR) (**Fig 1G and 1H**). The results indicated that IFN-α inhibits HCV replication in the S3-GFP replicon model but not in the R4-GFP cell line, but that IFN-λ1 inhibits HCV replication in both cell lines (S3-GFP and R4-GFP).

### IFN-λ activates the Jak–Stat signaling pathway in an IFN-α-resistant HCV cell culture

**Fig 2:**
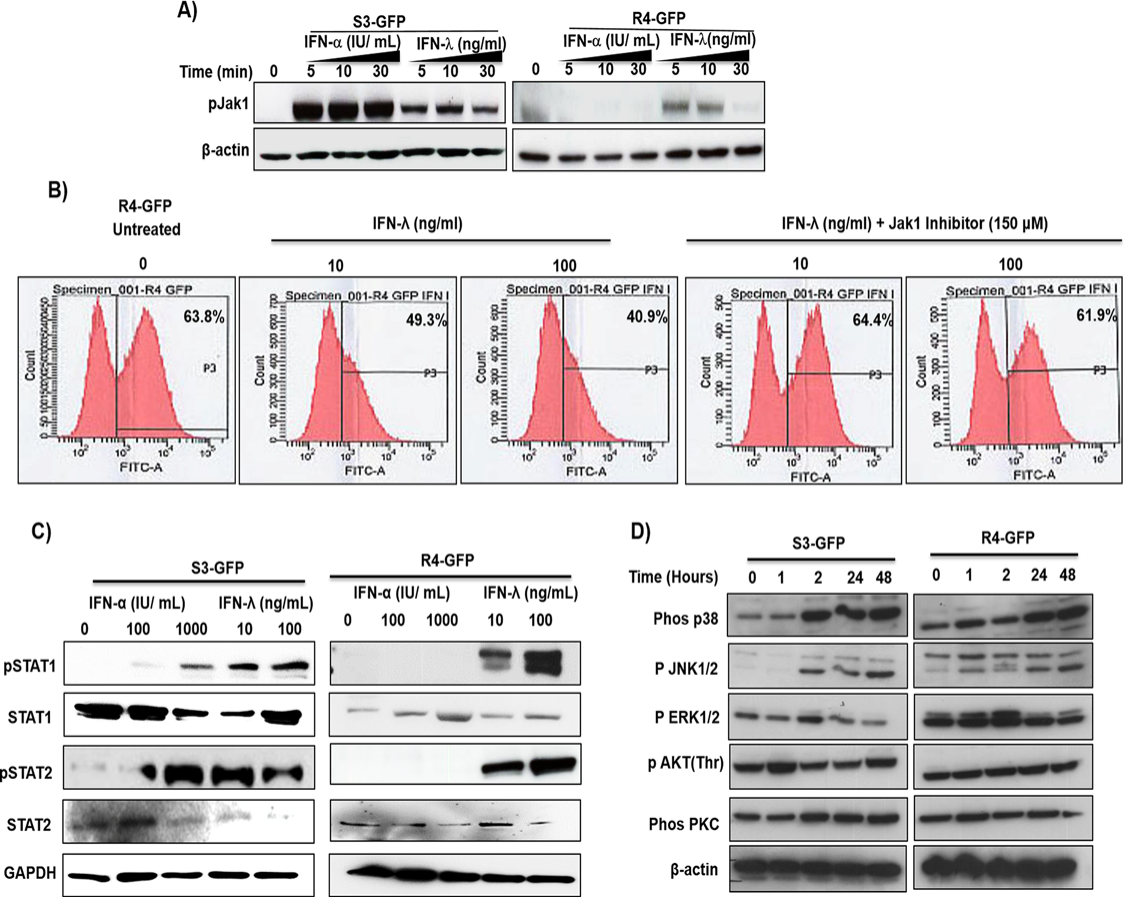
IFN-λ activates the JAK-STAT pathway in R4-GFP and S3-GFP cells. **(A)** R4-GFP and S3-GFP cells treated with IFN-α (1000 I.U/ml) or IFN-λ (100 ng/ml) for 5, 10 or 30 minutes and Jak phosphorylation was measured by Western blot analysis. **(B)** R4-GFP cells were treated with a pan-Jak inhibitor (6-Pyridone) for 1 hour prior to IFN-λ (100ng/mL) treatment and after 72 hours the antiviral effect was measured by FACs analysis. **(C)** STAT1 and STAT2 phosphorylation was determined by 30 minutes treatment of R4-GFP and S3-GFP cells with increasing doses of IFN-α or IFN-λ. **(D)** S3-GFP and R4-GFP cells were treated with 100ng/mL of IFN-λ and cells were collected at different time points after treatment. Western blotting was performed using antibodies targeting the p38, p-JNK, p-ERK and p-αKT and PKC and beta-αctin.

Binding of IFNs to receptors at the cell surface leads to activation of Jak family kinases, which then phosphorylate Stat proteins. The phosphorylated Stat proteins enter the nucleus and bind to specific DNA elements and thus direct ISG mRNA transcription. To determine if IFN-α or IFN-λ treatment results in activation of the Jak–Stat signaling pathway in R4-GFP cells, S3-GFP and R4-GFP cells were treated with IFN-α or IFN-λ1; then, phosphorylation of Jak1 was measured by Western blot analysis. Results of this experiment indicated that both IFN-α and IFN-λ induce Jak1 phosphorylation in the S3-GFP cell line, whereas only IFN-λ1 induces Jak1 phosphorylation in the R4-GFP cell line (**Fig 2A**). We then examined IFN-λ1 antiviral activity in R4-GFP cells in the presence and absence of pyridone-6, a Jak inhibitor, using flow analysis (**Fig 2B**). Pretreatment with Jak1 inhibitor prevented IFN-λ1 antiviral activity against HCV. To determine if IFN treatment could induce Stat 1 and Stat 2 phosphorylation, S3-GFP and R4-GFP cells were treated with equivalent concentrations of IFN-α or IFN-λ1 for 30 minutes. Cells were lysed and Stat phosphorylation was measured by Western blot analysis (**Fig 2C**). Both IFN-α and IFN-λ1 activated Stat 1 and Stat 2 phosphorylation in S3-GFP cells, whereas only IFN-λ1 induced phosphorylation of Stat 1 and Stat 2 in R4-GFP cells; IFN-α did not induce Stat 1 or Stat 2 phosphorylation in R4-GFP cells. We examined whether Type III IFNs also utilize an alternative circuit besides the Jak–Stat signaling pathway [31, 32]. The S3-GFP and R4-GFP cells were treated with IFN-λ1 and activation of different signaling pathways was examined by Western blot analysis. The results indicate that IFN-λ activates mitogen-activated protein kinase (MAPK) signaling and Akt phosphorylation in S3-GFP and R4-GFP cells (**Fig 2D**).

**Fig 3:**
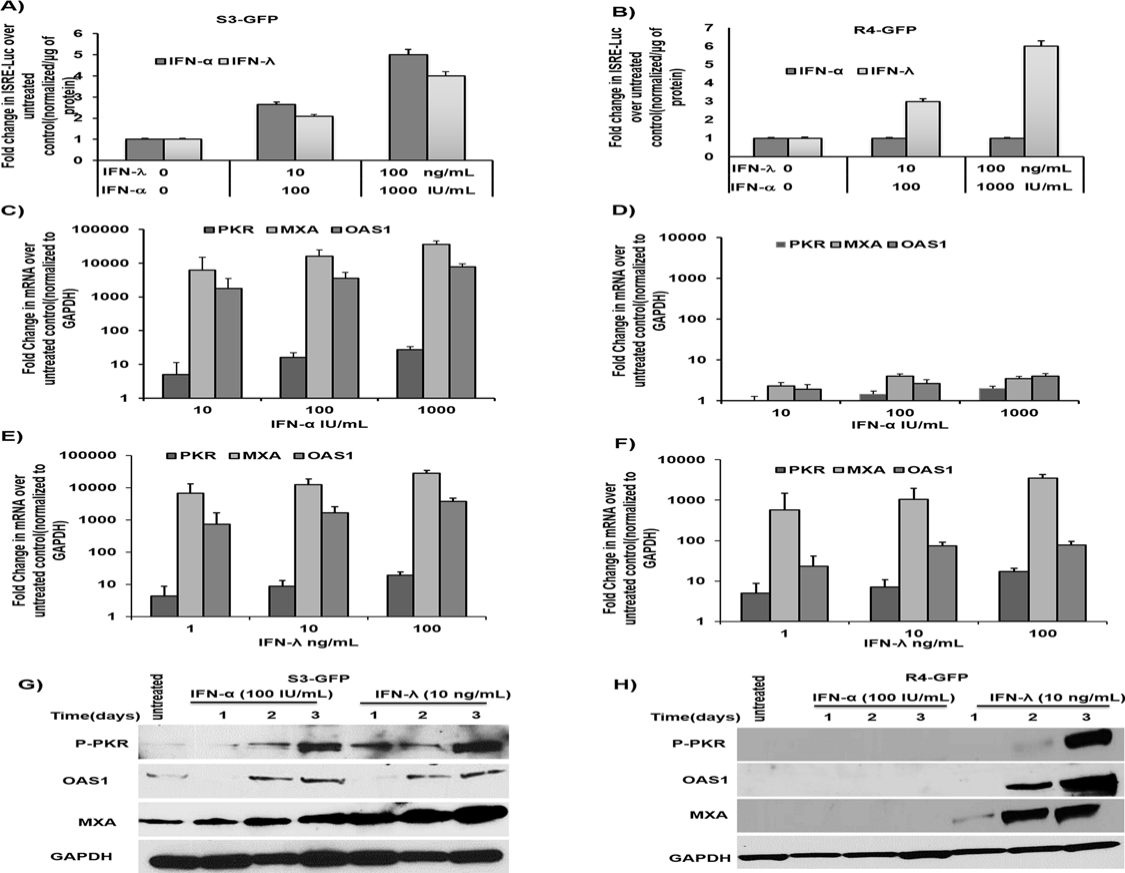
IFN-λ activates the ISRE promoter and induces antiviral ISGs in S3GFP and R4GFP cells. **(A)** S3-GFP cells were transfected with either an ISRE-Luc reporter plasmid or a control EGFP-Luc plasmid and treated with increasing doses of IFN-α or IFN-λ. Cells were collected 24 hours post transfection and Firefly luciferase activity was measured. Values were normalized with one microgram of protein and expressed as fold increase over the control. **(B)** R4-GFP cells were transfected with pISRE-Luc plasmid and treated with increasing doses of IFN-α or IFN-λ. After 24 hours, firefly luciferase activity was measured. **(C and D)** S3-GFP and R4-GFP cells were treated with increasing doses of IFN-α for 24 hours then the expression of ISGs mRNA level was quantified by qRT-PCR. The value of each sample was normalized to GAPDH and the expression levels relative to the untreated control were calculated. **(E and F)** S3-GFP and R4-GFP cells were treated with IFN-λ for 24 hours; the expression of ISG mRNA was measures by real-time RT-PCR. The value of each sample was normalized to GAPDH and the expression levels relative to the untreated control were calculated **(G and H)**. The protein levels of the ISGs were evaluated by Western blot in S3GFP and R4GFP cells treated with 100 I.U/mL of IFN-α or 10ng/mL of IFN-λ and collected every 24 hours for 72 hours.

It is known that a complex of phosphorylated Stat 1 and Stat 2 proteins plus IRF 9 enters the nucleus and binds to the interferon stimulated response element (ISRE) promoter of IFN-responsive genes to initiate antiviral gene transcription. We verified whether IFN-λ1 also activates ISRE-luciferase reporters in S3-GFP and R4-GFP cells using a transient transfection assay (**Fig 3A**); IFN-λ1 activated ISRE-luciferase expression in R4-GFP cells in a dose-dependent manner (**Fig 3B**). To verify that both IFN-λ1and IFN-α produce their antiviral activity through the induction of ISGs, we compared the induction of selected ISGs in S3-GFP and R4-GFP cells after IFN-α and IFN-λ1 treatment. The S3-GFP and R4-GFP cells were treated with increasing concentrations of IFN-λ1 or IFN-α, and tested for the activation of known ISGs such as PKR, MXA, and OAS 1; mRNA levels were measured by real-time quantitative RT-PCR. The IFN-α and IFN-λ1 induced expression of ISGs in S3-GFP cells in a dose-dependent manner (**Fig 3C and 3E**), whereas only IFN-λ treatment showed ISG induction in R4-GFP cells (**Fig 3D and 3F**). The induction of ISGs by both IFNs in S3-GFP and R4-GFP cell lines was verified by Western blot analysis (**Fig 3G and 3H**). Taken together, these analyses suggest that IFN-λ1 inhibits HCV replication in R4-GFP cells through activation of the Jak–Stat signaling pathway. These results are consistent with those of a previous report, indicating that IFN-λ1 utilizes the same Jak–Stat pathway as used by IFN-α to inhibit HCV replication in cell culture models [25].

### MicroRNA array provides evidence that IFN-λ decreases miR-122 transcription in R4-GFP cells

**Fig 4:**
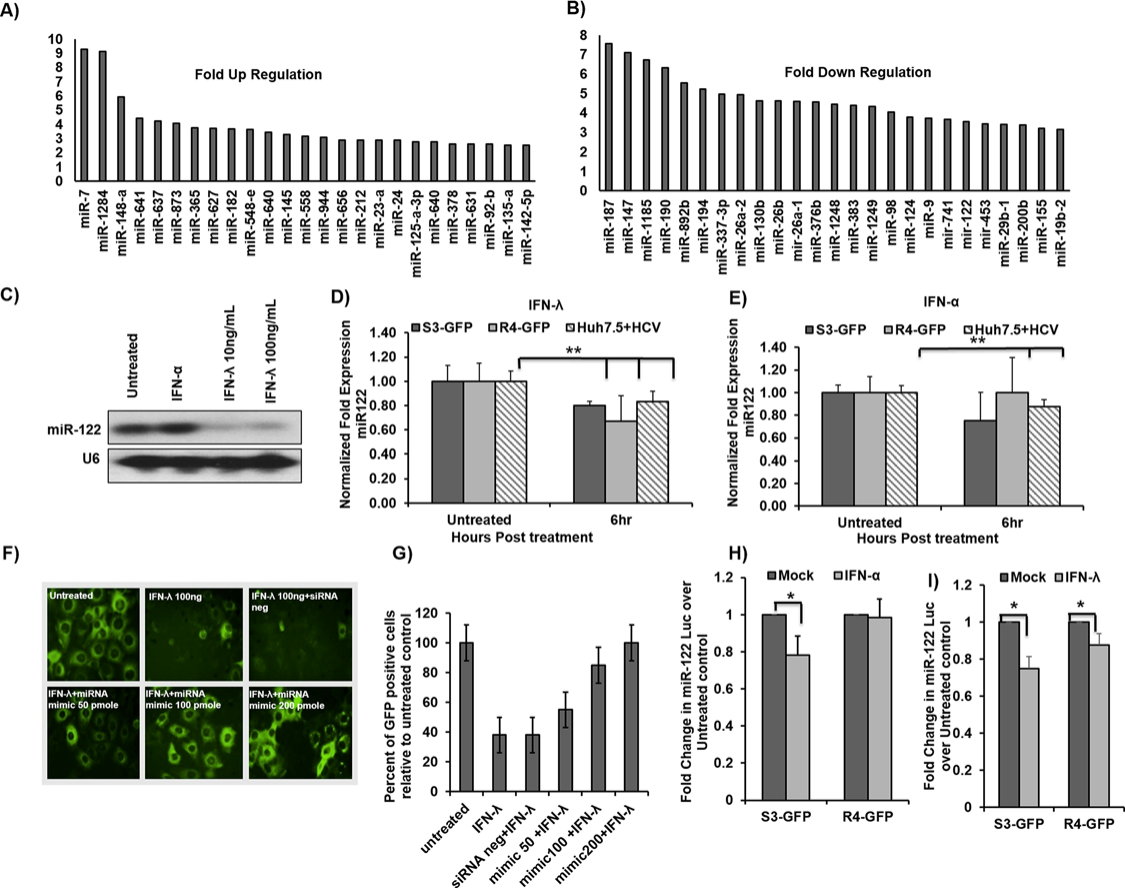
IFN-λ decreases miR-122 transcription in HCV cells. **(A)** Up regulated microRNAs expression in R4-GFP cells after IFN-λ treatment. **(B)** Down regulated MicroRNAs expression in R4-GFP cells after IFN-λ treatment. **(C)** Northern blot for miR-122 in R4-GFP cells treated with either IFN-α or IFN-λ for 6 hours. **(D and E)** S3-GFP, R4-GFP and persistently HCV infected Huh 7.5 cells were treated with either IFN-α (1000 IU/mL) or IFN-λ1 (100 ng/mL) for six hours. One microgram of total RNA was used for the measurement of miR-122 by real-time RT-PCR. **(F and G)** R4-GFP cells were transfected with increasing concentrations (25–100 pmole) of miR-122 mimic for 24 hours and then treated with IFN-λ for 48 hours. The effect of the mimic on the antiviral action of IFN-λ was assessed by fluorescence microscopy and then GFP positive cells were quantified by flow cytometry. **(H and I)** MiR-122-Luc promoter construct was transfected into S3-GFP and R4-GFP cells and following the transfection step, cells were treated with either IFN-α (1000 IU/mL) or IFN-λ (100ng/mL) for 6 hours. The effect of the treatment on the promoter activity was measured by comparing the fold change of firefly luciferase in the treated cells over the untreated control, *p≤0.05, **p≤0.001.

MicroRNAs are small non-coding RNA molecules that inhibit expression of target genes through a variety of mechanisms, by partially binding to complementary sites in specific messenger RNAs (mRNAs) through base pairing. MicroRNAs play a critical role in inflammation, interferon signaling, and antiviral mechanisms [33]. To understand their role in inducing HCV clearance by IFN-λ, we generated a microRNA profile for R4-GFP cells with and without IFN-λ1 treatment. This analysis revealed significant up-regulation and down-regulation of microRNA expression in R4-GFP cells after IFN-λ1 treatment (**Fig 4A and 4B, S1 and S2 Datasets**). The most interesting microRNAs are microRNA-122 (miR-122), and microRNA-24 (miR-24). MicroRNA-122 is expressed predominantly in the liver through multiple functions, including cholesterol metabolism, fatty acid biosynthesis, and support of HCV replication; it is known to be required for HCV replication, and our results described below show that HCV clearance by IFN-λ1 treatment is associated with a four-fold decrease in miR-122 levels in R4-GFP cells. The significance of our results is supported by previous studies that indicate that inhibition of miR-122 induces HCV clearance in chimpanzees [21]. The array results showing decreased expression of miR-122 expression in R4-GFP cells after IFN-λ treatment were confirmed by Northern blot analysis (**Fig 4C**); we found comparable miR-122 levels in untreated and IFN-α-treated R4-GFP cells. The decreased expression of miR-122 was quantified using real-time RT-PCR in S3-GFP cells, R4-GFP cells, and persistently HCV-infected cells after 6 hours of IFN treatment. Our analysis show that IFN-λ1 treatment significantly decreased miR-122 levels in S3-GFP, R4-GFP, and HCV-infected cells (**Fig 4D**), whereas IFN-α treatment decreased miR-122 levels in S3-GFP cells and infected cells (**Fig 4E**).

Another set of control experiments was performed to verify that miR-122 is involved in antiviral mechanisms of IFN-λ in an HCV cell culture model. We first determined whether over-expression of miR-122 mimic in R4-GFP cells could block the antiviral effect of IFN-λ. The R4-GFP cells were transfected with either miR-122 mimic or the scrambled oligonucleotide using Lipofectamine (Invitrogen). After 24 hours, R4-GFP cells were treated with IFN-λ1 (100 ng/mL) for an additional 72 hours. Anti-HCV effects were determined by fluorescence microscopy and then quantified by flow analysis. Results (**Fig 4F and 4G**) indicate that miR-122 mimic blocked the antiviral effect of IFN-λ1. To test the effect of IFN-λ1 on the miR-122 promoter, we used a luciferase plasmid containing the miR-122 promoter sequence, as described previously [34]; IFN-λ1 decreased the promoter activity of miR-122 in both S3-GFP and R4-GFP cells (**Fig 4I**), while IFN-α decreased the activity of the miR-122 promoter in S3-GFP only (**Fig 4H**). These results confirm that IFN-λ1 treatment decreased miR-122 transcription in R4-GFP cells.

### Decreased miR-122 transcription in IFN-λ-treated culture mediated by the HNF4α–Stat 3 feedback loop

**Fig 5:**
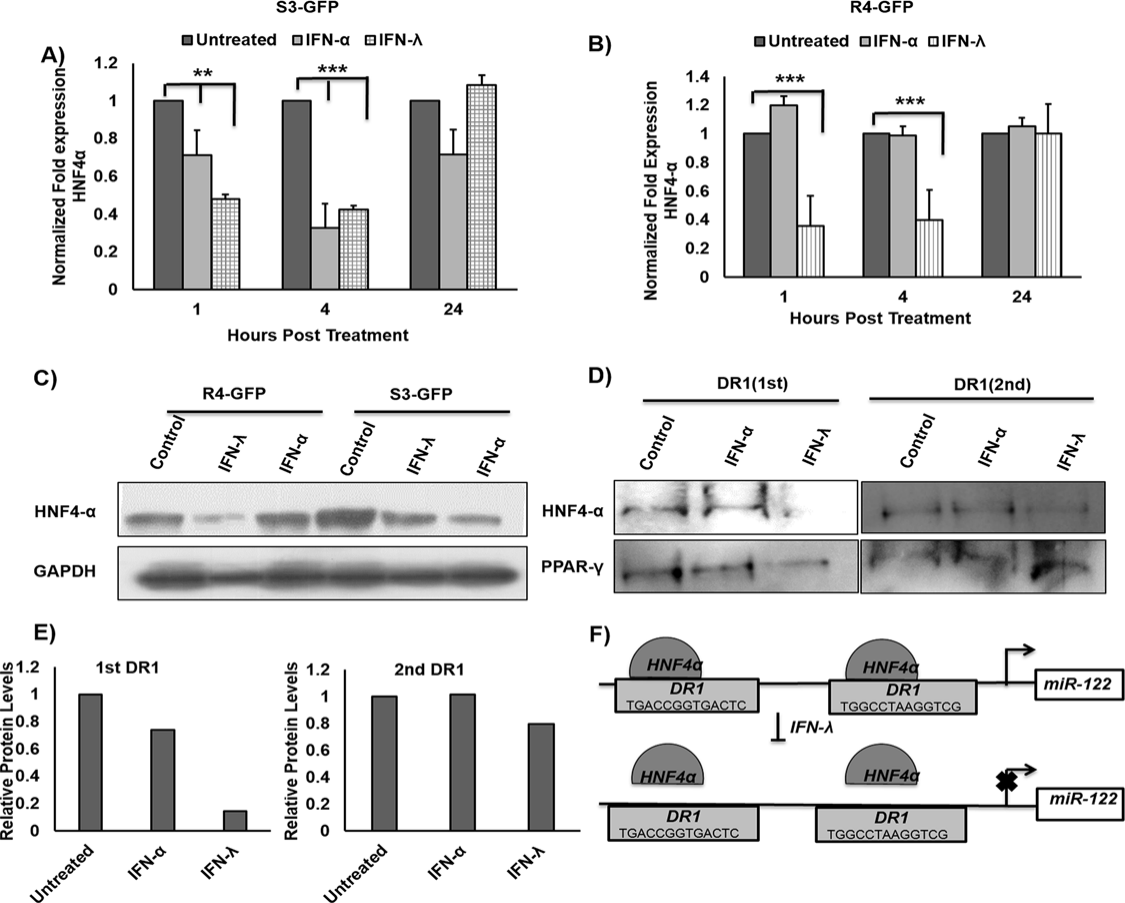
IFN-λ1 decreases miR-122 transcription through down regulation of HNF4α. **(A and B)** S3-GFP and R4-GFP cells were treated either IFN-α (1000 I.U/mL) or IFN-λ (100ng/mL). Cells were harvested at 1, 4 and 24 hours and then HNF4α mRNA levels were measured by real-time RT-PCR. **(C)** Protein levels of HNF4-α measured by Western blot in R4-GFP and S3-GFP after 4 hours of treatment with either 1000 IU/mL of IFN-α or 100ng/mL of IFN-λ. **(D)** Equal amount of cell lysates from R4-GFP cells were incubated with biotinylated double-stranded oligonucleotides corresponding to the two DR1 motifs in miR-122 promoter and with sterptavidin-agarose beads. The precipitated complexes were subjected to SDS-PAGE and Western blotting for HNF4α and PPAR-γ. **(E)** Quantification of the bands in Fig 5D **(F)** Schematic representation of putative HNF4α binding sites in human miR-122 gene promoters and the mechanisms for how IFN-λ1 decreases miR-122 transcription through reduced binding of HNF4α to DR1 element, **p≤0.01, ***p≤0.001.

Hepatic nuclear factor 4 alpha (HNF4α), a highly conserved member of the steroid/thyroid superfamily of transcription factors expressed in the liver, has been shown to positively regulate transcription by directly binding to miR-122 promoters [24]. To examine whether IFN treatment can reduce the expression of HNF4α, S3-GFP and R4-GFP cells were treated with IFN-α or IFN-λ for 4 hours, after which total RNA was isolated and HNF4α mRNA levels were quantified by real-time RT-PCR. As predicted, both IFNs inhibited HNF4α levels after 1 hour and 4 hours in S3-GFP cells (**Fig 5A**); however, HNF4α mRNA levels decreased in R4-GFP cells only after IFN-λ1 treatment (**Fig 5B**). Western blot analysis verified that IFN-λ1 inhibited HNF4α levels in R4-GFP cells, whereas both IFN-α and IFN-λ1 decreased expression of HNF4α in S3-GFP cells (**Fig 5C**). It has been shown that HNF4α binds to two core sites (DR1) in the miR-122 promoter [24]; thus, a DNA pull-down assay was performed to test if IFN-λ1 treatment decreased the binding of HNF4α at the DR1 sites in the miR-122 promoter. The IFN-λ1 treatment decreased the binding of HNF4α at both DR1 sites, as compared to the response in untreated R4GFP cells or cells treated with IFN-α (**Fig 5D**). To determine if this effect was specific to HNF4α, the same membrane was incubated with peroxisome proliferator-activated receptor gamma (PPARγ) antibody; PPARγ is another nuclear receptor known to bind to the DR1 sites in the miR-122 promoter [35]. The IFN-λ treatment did not decrease the binding of PPARγ to the DR1 sites (**Fig 5D and 5E**); however, IFN-λ1 treatment decreased expression of HNF4α, which resulted in reduced binding to the DR1 sites, leading to decreased transcription of miR-122.

The proposed mechanisms for decreased miR-122 expression by IFN-λ1 are summarized in **Fig 5F**. Of the microRNAs that are regulated by IFN-λ, Stat 3-inducible miR-24-5p shows a 2.1-fold up-regulation in our array (**Fig 4A**). Other laboratories have reported that the miR-24 promoter contains a putative Stat 3 binding site, allowing Stat 3 to induce miR-24 transcription, and that HNF4α expression is regulated by the binding of miR-24-3p to its 3′ untranslated region (3′UTR) [36].

**Fig 6:**
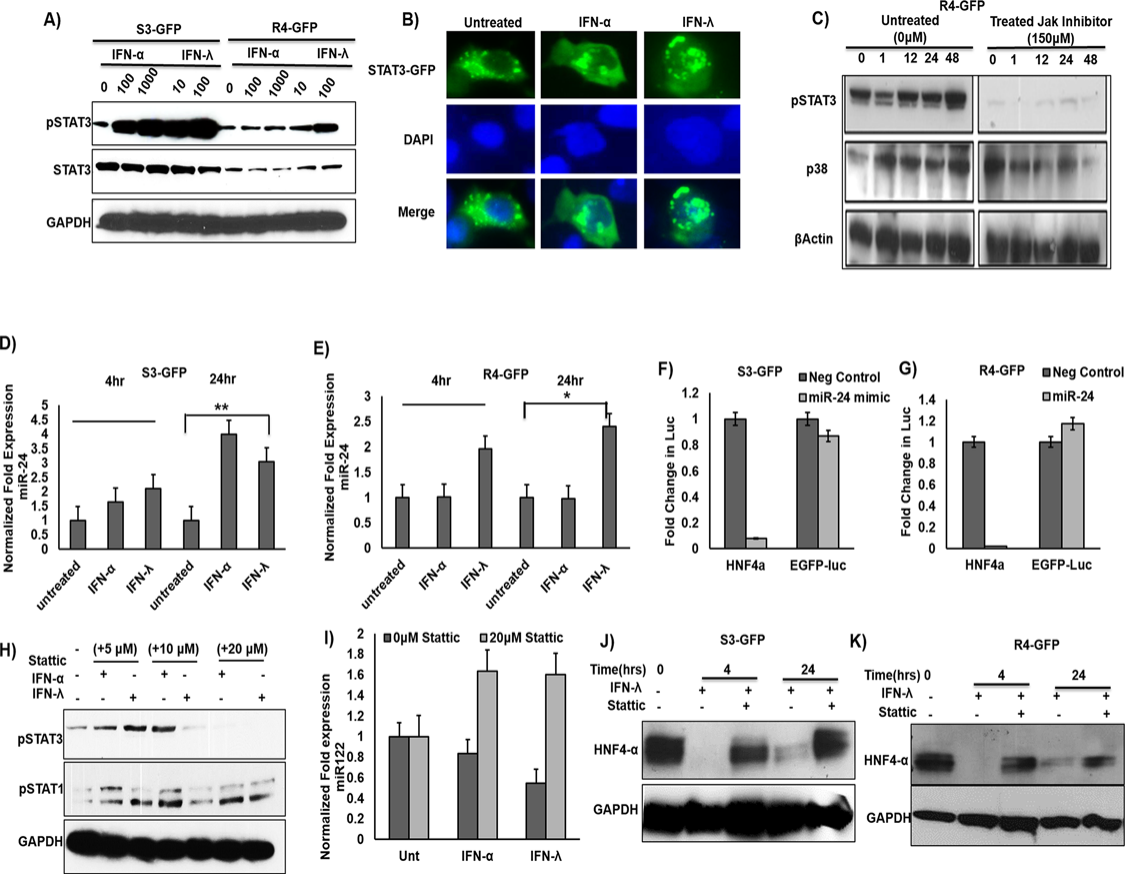
IFN-λ decreases HNF4α through STAT3 mediated expression of miR-24. **(A)** S3-GFP and R4-GFP cells treated with either IFN-α or IFN-λ for 30 minutes. Cell lysates were measured for pStat3 and total Stat3 activation by Western blot analysis. **(B)** Huh-7 cells were transfected with pSTAT3-GFP plasmid and cells were treated with either of IFN-α (1000 IU/mL) or IFN-λ (100ng/mL) for 1 hour. Nuclear translocation of the Stat3-GFP fusion protein was measured by fluorescence microscopy. **(C)** Treatment of R4-GFP cells with a Jak inhibitor prevented the effect of IFN-λ on STAT3 activation and but not the p38 activation. **(D and E)** S3-GFP and R4-GFP cells were treated with either with IFN-α (1000 IU/mL) or IFN-λ (100ng/mL). Quantification of miR-24 levels was done using qRT-PCR. **(F and G)** miR-24-3p mimic inhibited the luciferase activity of a reporter plasmid harboring the 3’UTR of HNF4α, while it had no effect on the control plasmid EGFP-Luc. **(H).** A STAT3 inhibitor (Stattic) specifically inhibits STAT3 phosphorylation in R4-GFP cells but has no effect on STAT1 phosphorylation. **(I)** Stattic prevents the down regulation of miR-122 by IFN-α and IFN-λ in S3-GFP cells 6 hours after treatment as determined by qRT-PCR. **(J and K)** Treatment of R4-GFP and S3GFP cells with Stattic prevented the inhibitory effect of IFN-λ on HNF4α, *p≤0.05, **p≤0.01.

Based on our array results, we hypothesize that Stat 3 activation by IFN-λ1 decreases transcription of miR-122 by inhibiting the expression of transcription factor HNF4α. To test this hypothesis, we examined the activation of Stat 3 in IFN-λ1 treated R4-GFP cells by Western blot analysis. As expected, a dose-dependent increase in Stat 3 levels was observed in both S3-GFP and R4-GFP cells after IFN-λ1 treatment (**Fig 6A**). To determine if IFN-λ1-induced-phosphorylated Stat 3 protein enhanced the nuclear translocation of Stat 3 in S3-GFP and R4-GFP cells, we transfected Stat 3-GFP plasmid Huh-7.5 cells and assessed nuclear translocation after IFN-λ1 treatment. The IFN-λ1 treatment activated Stat 3 and induced Stat 3-GFP translocation in uninfected Huh-7.5 cells (**Fig 6B**). Pre-treatment with Jak inhibitor blocked Stat 3 phosphorylation by IFN-λ in R4-GFP cells, but not p38 MAPK phosphorylation (**Fig 6C**). To determine if IFN-λ1 could increase transcription of miR-24 in HCV culture, S3-GFP and R4-GFP cells were treated with IFN-α or IFN-λ1 and the expression level of miR-24 was examined by real-time RT-PCR at 4 and 24 hours post-treatment (**Fig 6D**). Our results indicated that both IFN-α and IFN-λ induced expression of miR-24 levels in S3-GFP cells; however, only IFN-λ1 induced expression of miR-24 in R4-GFP cells (**Fig 6E**).To validate whether miR-24 can inhibit the 3’UTR of HNF4α in our cell lines, both S3-GFP and R4-GFP were transfected with a miR-24-3p mimic and a luciferase reporter plasmid harboring the 3’UTR of HnF4α. Indeed, the miR-24-3p decreased the luciferase activity of the HNF4α reporter plasmid in both cell lines, while it had no effect on the control plasmid. The results are expressed as the fold change in the luciferase activity compared to the negative control (**Fig 6F and 6G**).

**Fig 7:**
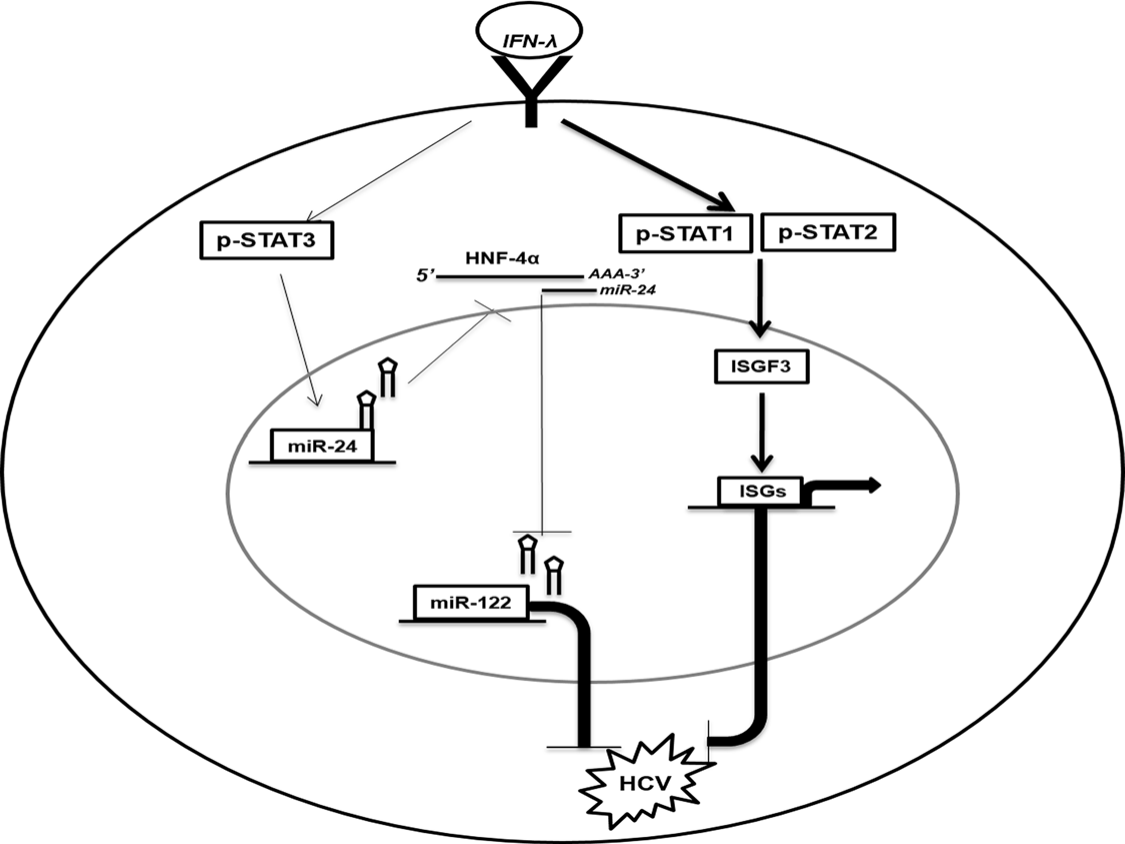
Schematic illustration of the anti-HCV mechanisms of IFN-λ involves two distinct pathways (The Jak-Stat and Stat3/HNF4α loop). IFN-λ first binds to cell surface receptor that activates the Stat1, Stat2 and Stat3 activation. **(i).** The Jak-Stat pathway activation involves the heterodimerization of Stat1/Stat2 which complexes with IRF9 to form ISGF3. The ISGF3 complex binds to the ISRE element to induce interferon stimulated gene expression and inhibit HCV. **(ii).** The Stat3 activation by IFN-λ induced the expression of miR-24. MiR-24 decreases HNF4α expression through binding to the 3’UTR of HNF4α mRNA. Reduced expression of HNF4α decreases the expression of miR-122 which inhibits HCV replication.

To determine if the activation of Stat 3 by IFN-λ-treated culture mediates the decreased expression of HNF4α, we measured HNF4α levels in R4-GFP cells in the presence and absence of the Stat 3 inhibitor Stattic, a small molecule know to specifically inhibit Stat 3 phosphorylation while having no effect on Stat 1 phosphorylation (**Fig 6H**). The S3GFP cells were pretreated with either Stattic or phosphate-buffered solution (PBS); 1 hour after treatment, the cells were treated with IFN-α or IFN-λ, and 6 hours after treatment, miR-122 levels were determined using quantitative RT-PCR. We were able to show that Stattic completely blocks the inhibitory effect of both IFNs on miR-122 (**Fig 6I**). Stat 3 inhibition also prevented IFN-λ1 mediated inhibition of HNF4α in R4-GFP and S3-GFP cells at the protein level (**Fig 6J and 6K**). Taken together, these results indicate that IFN-λ activates the Stat 3–HNF4α inflammatory circuit, in addition to the classical Jak–Stat antiviral pathway, thus leading to effective HCV clearance (**Fig 7**).

## Discussion

The purpose of this study was to understand the antiviral mechanisms by which IFN-λ1 inhibits HCV replication. Our results showed that IFN-λ1 (IL-29) strongly inhibits HCV replication in persistently infected HCV cell cultures, as well as in IFN-α-resistant replicon cell culture models. The R4-GFP sub-genomic cell line used in this investigation is an IFN-α-resistant replicon cell line, which does not respond to IFN-α treatment due to expression of a truncated IFNAR1 subunit. We found that IFN-λ1, a type III IFN, activates the classical Jak–Stat signaling pathway, inducing Stat 1, Stat 2, and Stat 3 phosphorylation, ISRE promoter activation, and ISG induction in IFN-α-sensitive as well as IFN-α-resistant cells. The IFN-λ1 induced anti-HCV effect is inhibited in R4-GFP replicon cells by the Jak inhibitor pyridone-6, indicating that the antiviral mechanism was initiated through the recruitment and activation of Jak kinases. These results are consistent with previous findings indicating that IFN-λ and IFN-α activate classical Jak–Stat signaling [22]. Chronic HCV infection impairs the IFN-α induced Jak–Stat signaling pathway through a variety of mechanisms [37]; IFN-λ1 induces a strong antiviral effect in the IFN-α-resistant replicon cell line, as well as in persistently infected HCV cultures. This finding is also consistent with observations made by other investigators, indicating that cross-regulation and sharing effects of type I and type III IFNs initiate Jak–Stat signaling [38, 39].

We found that in addition to Jak–Stat signaling, IFN-λ also activates other signaling pathways, such as MAPK, Stat 3, and Akt signaling, a finding that is consistent with the results of other investigators [31, 32]. IFN-λ activates Stat 3 in IFN-α-resistant and IFN-α-sensitive cell cultures. We focused our investigations on understanding the antiviral mechanism initiated by Stat 3 through regulation of microRNA expression. Stat 3 is an 89-kDa protein that is activated by a number of cytokines, including type I and type III IFNs. Interferon-activated Stat 3 can form a homo/hetero dimer, which can then translocate to the nucleus where it binds to DNA and initiates gene transcription [40]. Stat 3 activation has been linked to the expression of miR-24 and decreased activation of the inflammatory HNF4α loop. The miR-24 promoter contains highly conserved binding sites for Stat 3 [36]. MicroRNA is a small single-stranded RNA with 21–23 nucleotides that regulates gene expression by base pairing with complementary mRNA sequences to inhibit mRNA translation or induce mRNA degradation. MicroRNA-122 is a liver-specific microRNA that supports HCV replication through a variety of mechanisms, and its expression level has been implicated in IFN-α and RBV antiviral treatment [21–23]. Suppression of miR-122 has been linked to development of human hepatocellular carcinoma [41]. It is anticipated that microRNA can act either as an inducer or repressor of IFN-λ induced antiviral activity. The role of microRNAs in regulating IFN-λ antiviral activity is currently unknown.

To understand the role of microRNAs in IFN-λ antiviral activity against HCV, we performed a microRNA analysis of R4-GFP cells. Some microRNAs show a 2–9-fold increased expression, whereas expression of some microRNAs was reduced 2–7-fold after IFN-λ treatment. In our array results, we found that miR-122 expression is suppressed 3.55-fold. Our results revealed that Stat 3-inducible miR-24 expression is induced by IFN-λ1. MicroRNA-24 regulates the expression of HNF4α, which has been implicated in the transcriptional regulation of miR-122. Our array results motivated us to study the role of the Stat 3–HNF4α feedback loop in IFN-λ1 antiviral mechanisms. Consistent with the feedback-loop hypothesis, we found that IFN-λ1 activates Stat 3 phosphorylation and nuclear translocation, and induces expression of miR-24. Induced expression of miR-24 decreased expression of HNF4α and miR-122 transcription; this was confirmed by showing that a Stat 3 inhibitor could block HNF4α activation and miR-122 expression. Experiments in our laboratory have confirmed induction of Stat 3 by IFN-α and IFN-λ1 [42], yet the role of Stat 3 in the antiviral mechanisms has not been well established. Our results indicate that IFN-λ activates the HNF4α inflammatory loop to suppress miR-122 transcription. These results provide a potential antiviral mechanism involving activation of IFN-α and IFN-λ to inhibit HCV replication by suppression of miR-122 transcription.

Recently, it has been suggested that the Stat 3–HNF4α feedback loop is involved in inflammation and hepatic carcinogenesis [43]. Stat 3 activation has been implicated in the activation of this inflammatory loop in chronic liver disease (40). Stat 3 has been shown to be activated by the cytokine interleukin (IL-6) and by type I and type III IFNs. We have shown that the expression of IFN-α receptor-1 is impaired in chronic liver disease and liver cirrhosis due to ER stress and the autophagy response [29]. The expression of the IFN-λ receptor is restored in chronic liver disease as well as in cases of liver cirrhosis. The IFN-λ receptor is primarily expressed in epithelial cells, including in hepatocytes, but is absent in most hematopoetic cells [44]. In addition to IL-6, we have shown that IFN-λ also induces Stat 3 activation, indicating that IFN-λ activates this inflammatory circuit in the liver during chronic HCV infection. It has also been shown that HNF4α plays a role in the inflammation processes of the liver and hepatocellular carcinoma by regulating the transcription of miR-122 [44]. It has been reported that miR-122 loss leads to the development of hepatocellular carcinoma in knockout mice [41]; this implies that the Stat 3–HNF4α**-**mediated short-term suppression of miR-122 leads to viral clearance, whereas long-term activation of Jak–Stat signaling increases the risk of hepatocellular carcinoma. It has been claimed that IL-6-mediated activation of the Stat 3–HNF4α loop is inhibited by miR-124, which inhibits the IL-6 receptor. This study provides further evidence that IFN-λ activates the Stat 3–HFN 4α–miR-122 circuit independent of IL-6, which may be important for the development of hepatocellular carcinoma due to chronic inflammation.

Type I IFNs (IFN-α and IFN-β), type II IFNs (IFN-γ), and type III IFNs (IFN-λ1–3) levels are increased in cases of chronic liver disease; they are secreted by a wide variety of immune cells and are often produced by the activation of Toll-like receptors (TLRs) as well as cytosolic nucleic acid receptors (e.g., retinoic acid-inducible gene 1 and melanoma differentiation-associated gene 5) [20, 43]. Serum levels of IFΝ-λ also increase in chronic liver disease. The genetics of IFΝ-λ strongly influences the pathogenesis of chronic liver disease. Our results are supported by a clinical study based on 4172 patients, including those with chronic liver disease with viral and non-viral etiology, indicating that the IL-28B C/C genotype rs12970860 exhibits higher susceptibility to hepatic inflammation and fibrosis [7]. The group with the IL-28B C/C genotype responded well to IFN-α and RBV treatment. Basic research performed in our laboratory has shown that IL-28B C/C genotype hepatocytes respond well to IFN-λ treatment and induce Stat 1 and Stat 2 phosphorylation, whereas T/T hepatocytes are unable to induce Stat 2 phosphorylation and antiviral effects (44). Based on this evidence, we propose that short-term activation of the Stat 3–HNF4α loop activates antiviral signaling. However, long-term activation of this loop could increase the risk of inflammatory liver disease, liver cirrhosis, and hepatocellular carcinomas.

## Supporting Information

S1 Dataset Down regulated microRNA-R4-GFP+IFN-lambda. (XLS)

S2 Dataset Up-regulated mRNA-R4-GFP+IFNlambda. (XLS)
